# Functional Characterization of *Naematelia aurantialba* Basidiospore Polysaccharides in L929 Cells: Photoprotective, Antioxidant, and Anti-Inflammatory Effects Against UVB-Induced Damage

**DOI:** 10.3390/foods15030598

**Published:** 2026-02-06

**Authors:** Lihan Sun, Sijie Liu, Tao Sun, Rui Wang, Yian Gu, Liang Sun, Hong Xu, Peng Lei

**Affiliations:** 1 College of Food Science and Light Industry, Nanjing Tech University, No. 30 South Puzhu Road, Jiangbei New Area, Nanjing 211816, China; slh7062@163.com (L.S.); liusijie0813@163.com (S.L.); ruiwang2013@njtech.edu.cn (R.W.); yian.gu@hotmail.com (Y.G.); sunl@njtech.edu.cn (L.S.); xuh@njtech.edu.cn (H.X.); 2 Shenzhen Key Laboratory of Agricultural Synthetic Biology, Genome Analysis Laboratory of the Ministry of Agriculture and Rural Affairs, Agricultural Genomics Institute at Shenzhen, Chinese Academy of Agricultural Science, Shenzhen 518124, China; suntao05@caas.cn

**Keywords:** *Naematelia aurantialba*, basidiospore polysaccharides, functional characterization, UVB-induced damage, photoprotective, antioxidant, anti-inflammatory, L929 fibroblasts

## Abstract

Ultraviolet (UV) radiation is a primary driver of skin photoaging, characterized by oxidative stress, persistent inflammatory responses, and excessive degradation of the extracellular matrix (ECM). *Naematelia aurantialba* is a traditional medicinal and edible fungus recognized for its diverse pharmacological activities. In this study, *N. aurantialba* polysaccharides (NAPS-A)—high-value bioactive compounds obtained through liquid fermentation—were subjected to detailed functional characterization to evaluate their restorative potential against UVB-induced damage. The results demonstrated that NAPS-A treatment effectively mitigated UVB-induced cytotoxicity. Furthermore, NAPS-A significantly suppressed the accumulation of reactive oxygen species (ROS) and malondialdehyde (MDA), while robustly revitalizing the endogenous antioxidant defense system by restoring superoxide dismutase (SOD) and catalase (CAT) activities. Moreover, NAPS-A exerted potent anti-inflammatory effects by inhibiting the secretion of nitric oxide (NO) and pro-inflammatory cytokines, including IL-1β, IL-6, and TNF-α. NAPS-A maintained ECM homeostasis by counteracting collagen depletion, exhibiting inhibitory activity against collagenase and elastase, and modulating the mRNA expression of *Col1a1* and *Col3a1*. These findings suggested that NAPS-A protects fibroblasts from UVB-induced damage through a synergistic mechanism involving radical scavenging, the enhancement of cellular redox homeostasis, and the modulation of ECM metabolism. Overall, NAPS-A represents a promising, sustainably produced, food-derived bioactive ingredient with significant potential for the development of functional foods and nutricosmetics aimed at mitigating UVB-induced skin damage.

## 1. Introduction

Skin aging is a complex, multi-factorial biological process driven by both intrinsic (chronological) and extrinsic factors. Among environmental stressors, ultraviolet (UV) radiation is recognized as the predominant catalyst for extrinsic aging, a phenomenon termed photoaging [1,2,3]. Solar UV radiation reaching the Earth’s surface primarily comprises UVA (320–400 nm) and UVB (290–320 nm) [4]. Although UVB constitutes only a small fraction of total solar radiation, its higher photon energy exerts more profound biological effects on both epidermal and dermal cells. UVB can directly induce DNA photolesions, and produces large amounts of reactive oxygen species (ROS) and activate key transcription factors such as NF-κB and activator protein-1 (AP-1) [5,6]. The activation of these pathways promotes the secretion of pro-inflammatory cytokines and the upregulation of matrix metalloproteinases (MMPs), which accelerate collagen breakdown and structural remodeling of the dermis, thereby exacerbating the photoaging process. The accumulation of persistent photodamage serves as the fundamental pathological basis for skin photoaging. Therefore, effectively mitigating early-phase photodamage is not merely a protective measure but a critical intervention to prevent the progression toward permanent photoaged phenotypes.

In recent years, natural polysaccharides derived from edible and medicinal fungi have garnered significant interest as safe and effective bioactive agents, owing to their diverse pharmacological properties, including antioxidant, anti-inflammatory, and immunomodulatory effects [7,8,9]. For instance, polysaccharides isolated from *Tremella fuciformis*, *Ganoderma lucidum*, and *Pholiota nameko* have been shown to effectively mitigate UV-induced oxidative stress and preserve collagen integrity in dermal fibroblasts [10,11,12].

*Naematelia aurantialba* is a prestigious edible and medicinal basidiomycete fungus, widely utilized in traditional medicine in southwestern China [13]. Polysaccharides are considered the primary functional constituents of *N. aurantialba*, exhibiting potent free-radical scavenging and anti-inflammatory activities [14]. To circumvent the limitations of traditional extraction, liquid fermentation of *N. aurantialba* basidiospores has emerged as a robust technology to produce polysaccharides (NAPS-A) with high yields, consistent structural profiles, and excellent reproducibility. NAPS-A is a high-molecular-weight heteropolysaccharide (1.80 × 10^5^ to 1.35 × 10^6^ Da). Earlier research documented that NAPS-A predominantly characterized by an α-(1,3)-D-mannan backbone. This core framework is highly substituted with xylose, mannose, and glucuronic acid residues, where the β-D-Xylp branches are primarily attached at the O-2 and O-4 positions. These specific functional groups and its complex branched architecture are closely linked to the potent antioxidant and photoprotective capacities observed in this study [15,16].

Despite its potential, the specific photoprotective efficacy and the underlying mechanisms of NAPS-A against UVB-induced injury remain to be fully elucidated. In this study, we systematically investigated the protective effects of NAPS-A using a UVB-induced photodamaging model in L929 fibroblasts. We specifically focused on its ability to modulate oxidative stress, suppress inflammatory cascades, and regulate ECM metabolism, providing a scientific basis for the development of NAPS-A as a high-value, food-derived bioactive ingredient for functional food and nutraceutical applications.

## 2. Materials and Methods

### 2.1. Materials

Ethanol absolute, 3-(4,5-dimethylthiazol-2-yl)-2,5-diphenyltetrazolium bromide (MTT), fetal bovine serum (FBS), Dulbecco’s Modified Eagle Medium (DMEM), RPMI-1640 medium, Cell Counting Kit-8 (CCK-8), 2′,7′-dichlorodihydrofluorescein diacetate (DCFH-DA), and RIPA Lysis Buffer were purchased from Sigma-Aldrich Chemical Co. (St. Louis, MO, USA). Dimethyl Sulfoxide (DMSO) and 3-Amino,4-aminomethyl-2′,7′-difluorescein (DAF-FM DA) were purchased from Beyotime Biotechnology Co. (Shanghai, China).

### 2.2. Preparation and Purification of Polysaccharide from Naematelia aurantialba

*Naematelia aurantialba* strain NX-20 was obtained from the China General Microbiological Culture Collection Center with the unique identifier CGMCC 18588. Following a previously reported procedure [17], the NAPS fermentation broth was prepared. NAPS-A was extracted and purified following the protocols described by Sun [15], with its concentration quantified via the phenol-sulfuric acid method. The resulting NAPS-A samples were then lyophilized and kept in reserve for further studies.

### 2.3. Establishment of the UVB-Induced Photodamaging Model in L929 Cells

#### 2.3.1. Cell Culture

L929 fibroblast cells (CVCL_0462; ATCC, Manassas, VA, USA) were cultured in Dulbecco’s Modified Eagle Medium (DMEM) supplemented with 10% heat-inactivated fetal bovine serum (FBS) and 1% penicillin-streptomycin. The cells were maintained in a humidified incubator at 37 °C with an atmosphere of 5% CO_2_. For different experimental requirements, cells were seeded in 96-well plates (2.5 × 10^5^ cells/mL), 24-well plates (2.5 × 10^5^ cells/mL), or 6-well plates (4.0 × 10^5^ cells/mL) and allowed to adhere for 24 h before treatment.

#### 2.3.2. Cell Viability Assessment

Cell viability was assessed using the MTT assay to evaluate the cytocompatibility of NAPS-A. L929 cells were seeded in 96-well plates and treated with NAPS-A (dissolved in DMEM medium) at various concentrations (25, 50, 100, 200, and 500 μg/mL) for 24, 48, and 72 h. After the end of incubation, the medium was removed and wells were washed with PBS. Subsequently, 50 μL of MTT solution (1 mg/mL in PBS) was added to each well and incubated for 4 h at 37 °C. The MTT solution was removed, and the resulting formazan crystals (formed by viable cells) were subsequently dissolved in 100 μL of DMSO to act as a solubilizing solvent for optical density measurement. The absorbance was measured at 570 nm using a microplate reader (BioTek Synergy HT, Winooski, VT, USA). Cell viability was calculated as follows:

Cell viability = A_sample_/A_control_ (1)

where A_sample_ and A_control_ represent the absorbance of the NAPS-A treated group and the untreated control group.

#### 2.3.3. Collagenase and Elastase Activity Assays

Following 24 h of treatment with NAPS-A, L929 cells were harvested and lysed. The total protein concentration was determined using a BCA protein assay kit (Beyotime, Shanghai, China). The relative activities of collagenase and elastase were quantified using the EnzChek™ Collagenase and Elastase Assay Kits (Thermo Fisher Scientific, Waltham, MA, USA), respectively, according to the manufacturer’s protocols. Results were normalized to the total protein content of each sample.

#### 2.3.4. Dose Selection for UVB-Induced Photodamaging

UVB irradiation was performed using a UV-B lamp (TL40W/12RS; Philips; peak intensity of 312 nm). To determine the optimal dose, L929 cells were seeded in 96-well plates and starved in serum-reduced medium (DMEM with 3% FBS) for 12 h. Cells were then exposed to UVB radiation at doses of 0, 0.5, 1.0, 1.5, 2.0, and 2.5 mJ/cm^2^. During irradiation, the medium was temporarily replaced with a thin layer of phosphate-buffered saline (PBS). After exposure, PBS was replaced with fresh medium (3% FBS), and cells were incubated for an additional 24 h. Cell viability was then measured using the CCK-8 assay (450 nm) to determine the suitable irradiation dose for subsequent experiments.

#### 2.3.5. Data Normalization

To ensure accuracy across biochemical assays and account for UVB-induced cell loss, all physiological and biochemical parameters (e.g., ROS levels, enzyme activities) were normalized to either the total protein concentration (determined by BCA assay) or a fixed cell density (1.0 × 10^5^ cells) per sample.

### 2.4. Evaluation of the Restorative Effects of NAPS-A on UVB-Damaged Cells

#### 2.4.1. UVB Modeling and Group Assignment

To assess the repair potential of NAPS-A, L929 cells were cultured to reach confluence. Prior to irradiation, the growth medium was replaced with 100 μL of PBS. The experimental groups were defined as: (1) Control (CK), shielded with sterile aluminum foil; (2) UV, exposed to UVB radiation; and (3) NAPS-A Repair, exposed to UVB followed by NAPS-A treatment.

UVB irradiation was performed using a UV-B lamp (TL40W/12RS; Philips; peak intensity of 312 nm). The cells were exposed to a total dose of 1.5 mJ/cm^2^. The total radiant exposure (H) was calculated using the formula:

H (mJ/cm^2^) = P (mW/cm^2^) × t (s) (2)

where P represents the irradiance power and t is the exposure time.

Immediately following irradiation, PBS was removed and replaced with fresh DMEM containing NAPS-A at a final concentration of 50 μg/mL. The cells were then incubated for an additional 24 h to allow for cellular recovery and repair.

#### 2.4.2. Detection of Cell Apoptosis by AO/EB Staining

The impact of NAPS-A on cell viability following UVB injury was visualized using an Acridine Orange/Ethidium Bromide (AO/EB) Double Fluorescence Staining Kit (Bioss, Beijing, China). After the 24 h repair period, cells were washed twice with PBS and stained according to the manufacturer’s instructions. AO (green fluorescence) stains viable cells by intercalating into intact DNA, while EB (orange–red fluorescence) only penetrates cells with compromised membrane integrity, characteristic of apoptosis or necrosis. Stained cells were immediately observed and photographed using a fluorescence microscope (Olympus BX53, Tokyo, Japan).

### 2.5. Assessment of Oxidative Stress Defense

#### 2.5.1. Determination of Intracellular ROS Levels

The restorative effect of NAPS-A on UVB-induced oxidative stress was evaluated by quantifying reactive oxygen species (ROS) production. Intracellular ROS was detected using the 2′,7′-dichlorodihydrofluorescein diacetate (DCFH-DA) fluorescent probe (Beyotime, Shanghai, China). After the 24 h repair incubation, L929 cells were washed with PBS and incubated with 10 μM DCFH-DA at 37 °C for 20 min in the dark. The non-fluorescent DCFH-DA is oxidized by ROS to form highly fluorescent dichlorofluoroscein (DCF). The fluorescence intensity was quantified using a Varioskan Flash multimode microplate reader (Thermo Fisher Scientific, Waltham, MA, USA) at an excitation wavelength of 488 nm and an emission wavelength of 525 nm.

#### 2.5.2. Measurement of Oxidative Stress-Related Marker

To evaluate the capacity of NAPS-A to restore cellular redox homeostasis, the levels of malondialdehyde (MDA) and the activities of superoxide dismutase (SOD) and catalase (CAT) were quantified using commercial kits (Beyotime Biotechnology, Shanghai, China) according to the manufacturer’s instructions. After the 24 h repair treatment, L929 cells were harvested and lysed using ice-cold RIPA lysis buffer. The lysates were centrifuged at 12,000× *g* for 10 min at 4 °C, and the resulting supernatants were collected for subsequent biochemical assays.

MDA content was determined using the thiobarbituric acid (TBA) reactive substances assay. In brief, MDA in the samples reacts with TBA at high temperature (95 °C) and low pH to form a stable red-colored MDA-TBA adduct. The reaction mixture was cooled and centrifuged, and the absorbance of the supernatant was measured at 532 nm using a microplate reader.

Total SOD activity was determined using the WST-8 method. Superoxide anions (O_2_^−^) generated by the xanthine/xanthine oxidase system reduce WST-8 to form a water-soluble orange formazan dye. SOD scavenges O_2_^−^, thereby inhibiting the formation of formazan. The absorbance was measured at 450 nm after incubation at 37 °C for 20 min.

CAT activity was measured based on the rate of hydrogen peroxide (H_2_O_2_) decomposition. Residual H_2_O_2_ after the enzymatic reaction reacts with a chromogenic substrate (N-4-antipyryl-3-chloro-5-sulfonate-p-benzoquinonemonoimine) in the presence of peroxidase to produce a red product. The samples were incubated with 240 mM H_2_O_2_ at 25 °C for 5 min, followed by the addition of a stop solution. The absorbance was quantified at 520 nm using a microplate reader.

### 2.6. Determination of Intracellular NO Levels

The scavenging capacity of NAPS-A against intracellular nitric oxide (NO), a key inflammatory mediator, was evaluated using the DAF-FM DA fluorescent probe. DAF-FM DA is a cell-permeable, non-fluorescent compound that reacts with NO to form a stable, highly fluorescent benzotriazole derivative.

Following treatment, the cells were incubated with 5 μM DAF-FM DA (diluted in serum-free DMEM) at 37 °C for 20 min in the dark. After incubation, the cells were washed three times with PBS to remove excess extracellular probe. Fresh culture medium was then added, and the cells were incubated for an additional 15 min to ensure complete de-esterification of the intracellular diacetates. The fluorescence intensity, reflecting the intracellular NO concentration, was measured at excitation and emission wavelengths of 495 nm and 515 nm.

### 2.7. Determination of Pro-Inflammatory Cytokines

To evaluate the anti-inflammatory effect of NAPS-A following UVB exposure, the levels of key cytokines were measured. The culture supernatant was harvested and centrifuged at 11,180× *g* for 10 min at 4 °C to remove cellular debris. The concentrations of interleukin-6 (IL-6), interleukin-1β (IL-1β), and tumor necrosis factor-α (TNF-α) were quantified using mouse-specific enzyme-linked immunosorbent assay (ELISA) kits (Multi Sciences, Hangzhou, China) according to the manufacturer’s protocols. The absorbance was recorded at 450 nm using a microplate reader.

### 2.8. Quantification of Collagen Content

Following UVB exposure and subsequent 24 h treatment with NAPS-A, L929 cells were harvested and resuspended in 1 mL of cold 0.5 M acetic acid for lysis. The total collagen content was quantified using a Collagen Assay Kit (Sigma-Aldrich, St. Louis, MO, USA). Fluorescence intensity was measured at an excitation wavelength of 375 nm and an emission wavelength of 465 nm. The results were calculated based on a standard curve and normalized to the total protein content of the samples.

### 2.9. Gene Expression Analysis via RT-qPCR

The mRNA expression levels of collagen-related genes (*Col1a1* and *Col3a1*) and the pro-inflammatory gene *Tnf-α* were determined using quantitative reverse-transcription PCR (qPCR).

Total RNA was extracted from L929 cells using the RNAiso Plus kit (Takara Bio, Shiga, Japan). The purity and concentration of the RNA were assessed using a NanoDrop 2000 spectrophotometer (Thermo Fisher Scientific, Waltham, MA, USA). Subsequently, cDNA was synthesized using the PrimeScript™ RT Reagent Kit (Takara Bio, Japan) under the following conditions: 25 °C for 10 min, 42 °C for 15 min, and 85 °C for 5 min.

Real-time PCR was performed using SYBR^®^ Premix Ex Taq™ II (Takara Bio, Japan) on a QuantStudio 3 Real-Time PCR System (Thermo Fisher Scientific, Waltham, MA, USA). *GAPDH* was employed as the internal reference gene. The relative mRNA expression levels were calculated using the 2^−ΔΔCt^ method. The primer sequences used are summarized in Table 1.

Relative Gene Expression = 2^−ΔΔCt^

where ΔCt = Ct (target gene) − Ct (reference gene), ΔΔCt = ΔCt (treatment) − ΔCt (control).

### 2.10. Statistical Analysis

All data were obtained from at least three independent experiments and are presented as mean ± SD. Statistical analyses were performed using SPSS Statistics software (version 31.0; IBM, Armonk, NY, USA).

For comparisons among multiple groups, one-way analysis of variance (ANOVA), followed by Tukey’s honestly significant difference (HSD) post hoc test, was applied. Comparisons between two groups were conducted using a *t*-test. A *p*-value < 0.05 was considered statistically significant.

## 3. Results

### 3.1. Effect on Cell Viability

Ensuring the absence of cytotoxicity is a fundamental prerequisite for the development of bioactive compounds intended for dermatological and cosmetic applications. The cytocompatibility of L929 fibroblasts treated with NAPS-A was systematically evaluated at concentrations ranging from 25 to 500 μg/mL over 24, 48, and 72 h.

**Figure 1:**
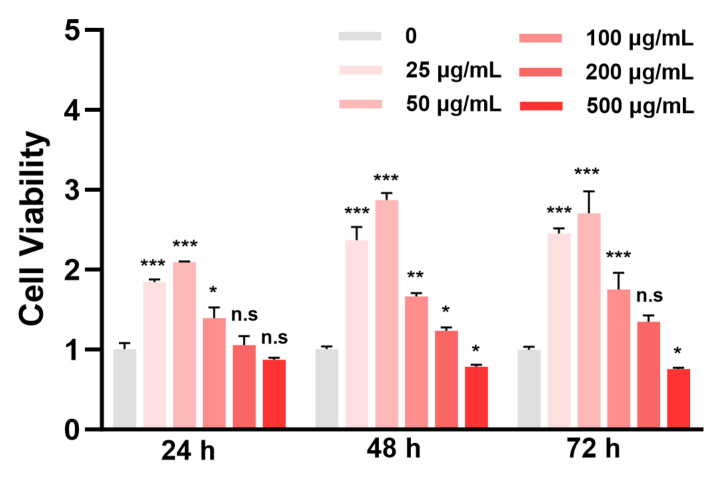
Cell viability after treatment with various concentrations of NAPS-A (25–500 μg/mL) for 24, 48, and 72 h, measured by the MTT assay. Data are presented as mean ± SD (*n* = 3). * *p* < 0.05, ** *p* < 0.01, and *** *p* < 0.001 indicate statistical significance compared to untreated control group; n.s. indicates no significant difference (*p* > 0.05) compared to the untreated control group.

As shown in Figure 1, no significant reduction in cell viability was observed at concentrations up to 200 μg/mL throughout the 72 h experimental duration (*p* > 0.05), indicating that NAPS-A exhibited good cytocompatibility within this concentration range. Thus, the non-cytotoxic NAPS-A concentration with a limit of 200 µg/mL were chosen for L929 cell treatment in subsequent experiments. Moreover, NAPS-A promoted L929 fibroblasts viability, but only within the concentration range of 25–200 μg/mL.

### 3.2. Inhibitory Effects of NAPS-A on Collagenase and Elastase Activities

The integrity of the extracellular matrix (ECM) is primarily maintained by structural proteins such as collagen and elastin [18]. Collagenase and elastase are the hallmark enzymes responsible for the degradation of these proteins, and their overactivation is a key driver of wrinkle formation and loss of skin elasticity in photoaging [19].

**Figure 2:**
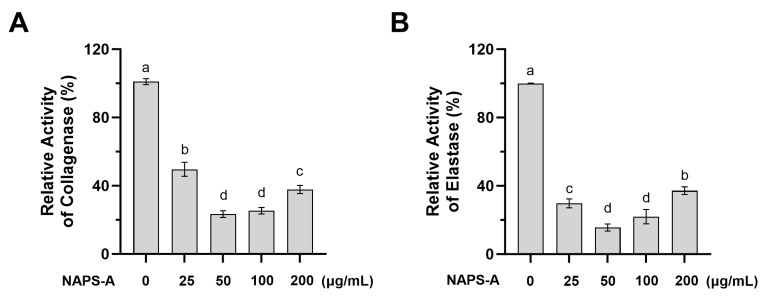
Inhibitory effects of NAPS-A on collagenase and elastase activities. (**A**) Relative activity of collagenase in L929 fibroblasts treated with NAPS-A (0–200 μg/mL) for 24 h; (**B**) Relative activity of elastase in L929 fibroblasts treated with NAPS-A (0–200 μg/mL) for 24 h. Each bar represents the mean ± SD (n = 3). Values with different letters are significantly different from each other (*p* < 0.05).

As illustrated in Figure 2, at the tested concentrations, NAPS-A significantly inhibited both collagenase and elastase activities compared to the control group (*p* < 0.05). This inhibitory effect exhibited clear dose-dependency within the concentration range of 0–50 μg/mL.

### 3.3. Restorative Effects of NAPS-A Against UVB-Induced Photodamage

**Figure 3:**
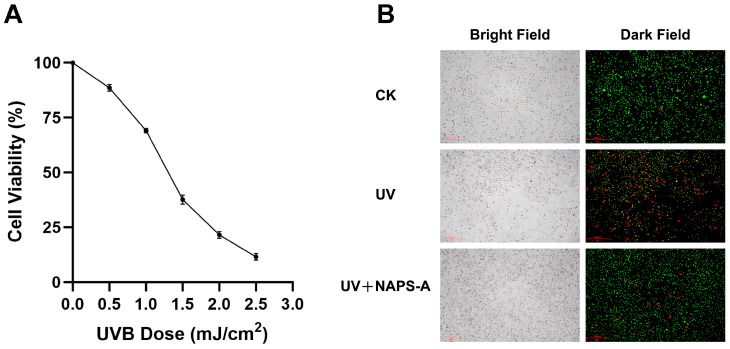
Restorative Effects of NAPS-A against UVB-Induced photodamage. (**A**) Effect of varying UVB irradiation doses on the viability of L929 cells. Data are presented as mean ± SD (*n* = 3). (**B**) The representative fluorescence micrographs show L929 cells from the control (CK), UVB-irradiated (UV), and UVB + 50 μg/mL NAPS-A groups after AO/EB staining. Viable cells are indicated by green fluorescence, while non-viable (dead) cells are indicated by orange–red fluorescence, as marked by the red arrows.

To establish a photodamaging model, L929 fibroblasts were exposed to varying doses of UVB radiation. As shown in Figure 3A, UVB irradiation led to a significant, dose-dependent decrease in cell viability (*p* < 0.05). A dose of 1.5 mJ/cm^2^ reduced cell viability to 37.62% and was therefore chosen as the optimal dose for subsequent repair experiments. This choice aligned with the established in vitro model of UVB-induced photodamaging in fibroblasts [11].

The restorative potential of NAPS-A was further validated through AO/EB dual fluorescence staining (Figure 3B). In the UVB-irradiated model group, a dramatic increase in orange–red fluorescence was observed, indicating compromised membrane integrity and the onset of apoptosis. In contrast, post-treatment with NAPS-A (50 μg/mL) markedly reduced the proportion of EB-positive (orange-red) cells and restored the predominance of AO-positive (bright green) cells with normal spindle-shaped morphology. These results visually demonstrated that NAPS-A effectively intervenes in the cell death process and facilitates the recovery of L929 fibroblasts from UVB-induced lethal injury.

### 3.4. NAPS-A Attenuates UVB-Induced Oxidative Stress and Restores Redox Homeostasis

The excessive accumulation of reactive oxygen species (ROS) is a hallmark of skin photodamaging. UVB irradiation triggers the overproduction of intracellular ROS, which disrupts the cellular redox balance, leading to oxidative damage of lipids, proteins, and DNA, and eventually culminating in cell death [20].

#### 3.4.1. Scavenging of Intracellular ROS

**Figure 4:**
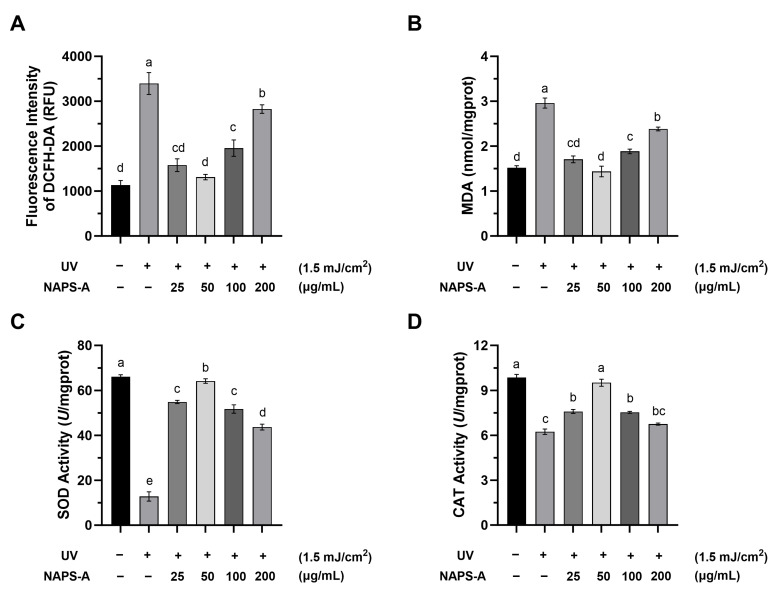
NAPS-A mitigates UVB-induced oxidative stress and restores antioxidant enzyme activities. L929 cells were irradiated with 1.5 mJ/cm^2^ UVB followed by treatment with NAPS-A (25–200 μg/mL) for 24 h: (**A**) intracellular ROS levels (expressed as DCFH-DA fluorescence intensity); (**B**) MDA content; (**C**) SOD activity; (**D**) CAT activity. “+” and “−” indicate the presence or absence of UV irradiation or NAPS-A treatment, respectively. Values represent the mean ± SD (*n* = 3). Values with different letters are significantly different from each other (*p* < 0.05).

As illustrated in Figure 4A, UVB irradiation significantly elevated intracellular ROS levels by 2.99-fold in the model group compared to the control (*p* < 0.05). Post-treatment with NAPS-A effectively quenched this oxidative burst. At concentrations of 25, 50, 100, and 200 μg/mL, NAPS-A reduced ROS levels to 46.46%, 38.66%, 57.60%, and 83.26% of the model group, respectively. The most robust scavenging activity was observed at 50 μg/mL. This antioxidant efficacy may be attributed to the presence of glucuronic acid and specific hydroxyl groups in NAPS-A, which can directly neutralize free radicals and terminate oxidative chain reactions [21].

#### 3.4.2. Mitigation of Lipid Peroxidation (MDA)

Malondialdehyde (MDA) is a primary byproduct of lipid peroxidation and serves as a critical biomarker for assessing the severity of oxidative injury [22]. Following NAPS-A treatment (25–200 μg/mL), the MDA content in UVB-photodamaged L929 cells was significantly reduced to 60.09%, 44.33%, 64.94%, and 76.05% of the model group, respectively (Figure 4B). Consistent with the ROS data, the most significant inhibition was achieved at 50 μg/mL.

#### 3.4.3. Restoration of Endogenous Antioxidant Enzyme Activities (SOD and CAT)

Superoxide dismutase (SOD) and catalase (CAT) constitute the first line of the cellular enzymatic defense system, responsible for converting superoxide anions into water and oxygen. Compared to the control group, the activities of both SOD and CAT were markedly suppressed in the UV group (Figure 4C,D), indicating a collapse of the endogenous antioxidant system.

NAPS-A treatment significantly revitalized these enzymes. Notably, at the optimal concentration of 50 μg/mL, NAPS-A elevated SOD and CAT activities by 5.00-fold and 1.52-fold relative to the model group, respectively.

### 3.5. NAPS-A Mitigates UVB-Induced Inflammatory Responses

UVB-induced oxidative stress is inextricably linked to the activation of inflammatory cascades. This process is characterized by the overproduction of nitric oxide (NO) and the release of key pro-inflammatory cytokines, which further exacerbate cellular damage and extracellular matrix degradation [23].

#### 3.5.1. Suppression of NO and Pro-Inflammatory Cytokine Secretion

**Figure 5:**
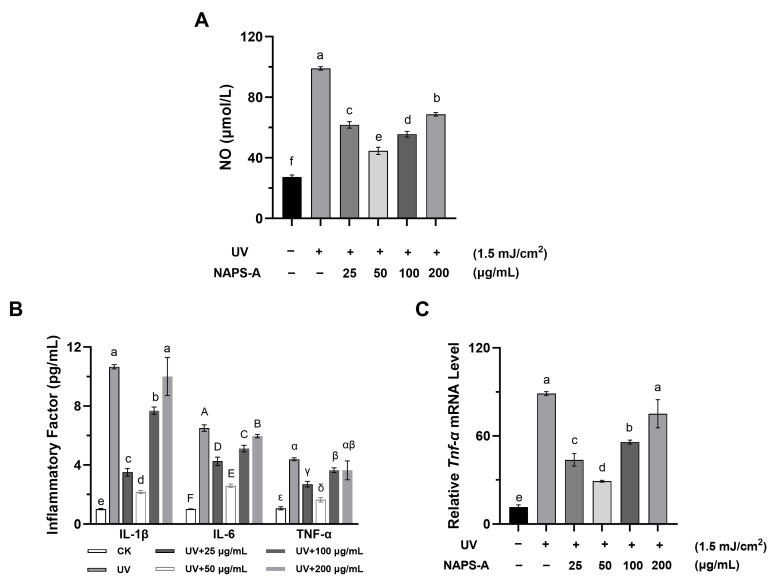
Anti-inflammatory effects of NAPS-A on UVB-irradiated fibroblasts: (**A**) NO production; (**B**) IL-1β, IL-6 and TNF-α secretion; (**C**) relative mRNA expression of *Tnf-α*. “+” and “−” indicate the presence or absence of UV irradiation or NAPS-A treatment, respectively. Cytokine levels were measured by ELISA and gene expression was measured by RT-qPCR. Data are mean ± SD (*n* = 3). Values with different letters are significantly different from each other (*p* < 0.05).

As shown in Figure 5A, UVB irradiation triggered a significant surge in NO production, a reactive signaling molecule that contributes to oxidative injury. Treatment with NAPS-A effectively curtailed this increase, restoring NO to near-basal levels. Furthermore, ELISA analysis revealed that the secretion of major pro-inflammatory cytokines—IL-1β, IL-6, and TNF-α—was markedly elevated following UVB exposure (Figure 5B). NAPS-A treatment significantly suppressed the release of these mediators, indicating its potent anti-inflammatory activity during the post-irradiation recovery phase.

#### 3.5.2. Downregulation of *Tnf-α* Gene Expression

To further confirm these findings at the transcriptional level, RT-qPCR was performed to assess the mRNA levels of *Tnf-α*. Consistent with the protein secretion data, UVB irradiation induced a robust upregulation of *Tnf-α* transcripts, whereas NAPS-A treatment significantly downregulated its expression (Figure 5C). Within the concentration range of 25–50 μg/mL, NAPS-A exhibited a dose-dependent inhibitory effect on *Tnf-α* expression in UVB-irradiated L929 cells. Notably, the most significant suppressive efficacy was observed at 50 μg/mL, indicating that this concentration serves as an optimal threshold for NAPS-A to mitigate UVB-induced inflammatory responses.

### 3.6. NAPS-A Promotes the Recovery of Collagen and ECM Integrity

The degradation of the extracellular matrix (ECM), particularly the loss of structural collagen, is the primary pathological feature of skin photoaging [24]. To evaluate the efficacy of NAPS-A in reversing this damage, we analyzed both total collagen content and the transcriptional levels of major collagen types.

#### 3.6.1. Restoration of Intracellular Collagen Content

**Figure 6:**
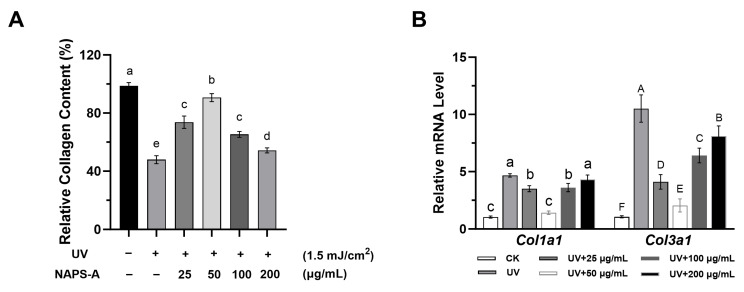
NAPS-A promotes collagen synthesis and ECM integrity: (**A**) intracellular collagen content; (**B**) relative mRNA expression of *Col1a1* and *Col3a1*. “+” and “−” indicate the presence or absence of UV irradiation or NAPS-A treatment, respectively. All gene expression data were normalized to *GAPDH* using the 2^−ΔΔCt^ method. Results are shown as mean ± SD (*n* = 3). Values with different letters are significantly different from each other (*p* < 0.05).

As shown in Figure 6A, UVB irradiation significantly depleted the intracellular collagen levels in L929 fibroblasts, with the most pronounced efficacy observed at a concentration of 50 μg/mL, reflecting the catastrophic breakdown of the ECM framework. However, treatment with NAPS-A successfully restored collagen content, significantly increasing it compared to the UVB-irradiated model group (*p* < 0.05).

#### 3.6.2. Regulation of *Col1a1* and *Col3a1* Gene Expression

To explore the response of extracellular matrix (ECM) components to UVB and NAPS-A, the mRNA expressions of type I (*Col1a1*) and type III (*Col3a1*) collagen were quantified. As shown in Figure 6B, UVB exposure led to a significant upregulation of *Col1a1* and *Col3a1* transcription compared to the control group. Interestingly, the upregulation of collagen genes (e.g., *Col1a1* and *Col3a1*) suggests that L929 cells may initiate a compensatory response to replenish the collagen degraded by UVB radiation [25]. NAPS-A treatment was found to modulate these expression levels, demonstrating its ability to alleviate UVB-induced ECM impairment. By mitigating the primary damage, NAPS-A relieved the cells from the necessity of excessive gene upregulation to maintain ECM proteostasis.

## 4. Discussion

In this study, we successfully utilized liquid fermentation of *Naematelia aurantialba* basidiospores to isolate NAPS-A. By employing a UVB-induced injury model in L929 fibroblasts, we demonstrated that NAPS-A serves as a potent restorative agent, providing a theoretical framework for its application as a natural anti-photoaging ingredient.

In the cell viability assay, NAPS-A treatment at certain concentrations resulted in absorbance values significantly higher than those of the control group. This phenomenon suggested that NAPS-A actively stimulated the metabolic activity or proliferation of L929 fibroblasts. Similar polysaccharides derived from the medicinal mushroom *Auricularia auricula-judae* have been reported to exhibit a comparable stimulatory effect on fibroblast proliferation [26].

A hallmark of dermal aging is the catastrophic degradation of the extracellular matrix (ECM) [27]. Our findings revealed that NAPS-A significantly enhances intracellular collagen content and modulates the transcriptional expression of *Col1a1* and *Col3a1*. In our study, an unexpected increase in *Col1a1* and *Col3a1* mRNA levels was observed following UVB irradiation. While chronic UVB exposure typically leads to collagen deficiency, acute exposure can trigger an adaptive compensatory mechanism where the cells attempt to offset protein degradation by temporarily boosting gene transcription. This phenomenon aligned with the “stress-induced compensation” theory observed in early-stage dermal damage [25]. Although the direct signaling pathways were not investigated in this study, the restoration of redox balance and the suppression of inflammatory cytokines by NAPS-A may contribute to a favorable microenvironment for collagen maintenance. Previous literature suggests that such effects are often mediated via the TGF-β/Smad or MAPK pathways [28]. Our results provided a strong pharmacological basis for NAPS-A as a protective agent, though further studies are required to elucidate the specific molecular docking and intracellular signaling targets involved.

Moreover, NAPS-A (50 μg/mL) displayed robust inhibitory activities against collagenase (49.63%) and elastase (29.74%), significantly outperforming *Pleurotus ostreatus* polysaccharide fractions. For instance, the POP-40 fraction was reported to achieve only 19.82% collagenase inhibition at a higher dose (62.5 μg/mL), while the maximum elastase inhibition for POP-60 reached merely 24.54% even at 1.0 mg/mL [29]. The superior efficacy of NAPS-A at lower concentrations highlighted its potential as a high-efficiency collagenase and elastase inhibitor. This potent bioactivity was likely attributed to its unique α-(1,3)-D-mannan branching patterns and the enrichment of glucuronic acid residues. These structural moieties may influence matrix metalloproteinases (MMPs), thereby attenuating ECM degradation and preserving the structural integrity of the skin [30].

The “Oxygen Stress Theory” of aging posits that cumulative oxidative damage is the primary driver of cellular senescence [31]. NAPS-A treatment not only directly scavenged ROS and MDA but also revitalized the endogenous antioxidant defense system by upregulating SOD and CAT activities. In summary, NAPS-A enhances cellular antioxidant capacity by maintaining the structural and functional integrity of key enzymes. The monosaccharide profile of *N. aurantialba* polysaccharides—comprising D-mannose, D-xylose, and D-glucuronic acid—likely underpins these bioactivities. These acidic components may trigger intracellular signaling pathways, such as the Nrf2/ARE pathway, to upregulate the expression of antioxidant genes, thereby providing a sustained defense against UV-induced damage [32,33]. Interestingly, a non-linear, bell-shaped dose–response relationship was observed, with the maximal restorative efficacy achieved at 50 μg/mL. Similar dose-dependent patterns have been frequently reported for natural polysaccharides [34]. At higher concentrations, increased solution viscosity and steric hindrance may limit effective interactions with reactive oxygen species, while cellular uptake and antioxidant signaling pathways may reach a saturation threshold [35,36]. Consequently, these factors may collectively attenuate antioxidant efficiency at doses exceeding the optimal range.

Furthermore, NAPS-A significantly suppressed the secretion of pro-inflammatory cytokines (IL-1β, IL-6, and TNF-α) and reduced NO production. Previous studies have indicated that inhibiting the NF-κB/NLRP3 inflammasome can significantly alleviate cutaneous inflammation and prevent premature aging [37,38]. Given our results, we hypothesized that NAPS-A exerts its anti-inflammatory action by modulating this axis, possibly by preventing the nuclear translocation of NF-κB or the assembly of the NLRP3 inflammasome. This provided a promising direction for future mechanistic studies using advanced molecular docking and protein–protein interaction assays.

While the current data provided compelling evidence for the bioactivity of NAPS-A, it was essential to evaluate the inherent strengths and potential constraints of this study. This study provided the first systematic functional characterization of NAPS-A, a unique polysaccharide derived from the basidiospores of *Naematelia aurantialba* via liquid fermentation. A major strength lay in the identification of its multi-target UVB-protective efficacy, which simultaneously addressed oxidative stress, inflammatory cascades, and ECM metabolism in UVB-irradiated L929 fibroblasts. Unlike many botanical extracts, NAPS-A exhibited high potency at relatively low concentrations (50 μg/mL), outperforming several known fungal polysaccharides in enzyme inhibition.

Despite these promising findings, several limitations remain to be addressed. First, while the current study focused on biochemical and cellular markers, future validation utilizing more sophisticated diagnostic tools—such as HPLC-TBARS for precise lipidomic profiling and Laser Doppler flowmetry for assessing microvascular responses—will be essential to fully align these in vitro findings with clinical skin physiological outcomes [39]. Second, although we observed significant modulation of key biomarkers like ROS and TNF-α, the precise intracellular signaling pathways (e.g., Nrf2/ARE or NF-κB) through which NAPS-A exerts its effects remain to be definitively elucidated via transcriptomic or Western blot analysis. Nevertheless, in vivo validation using appropriate animal models will be essential to further confirm the efficacy and safety of NAPS-A and to assess its translational potential for dermatological applications. Addressing these gaps will be the focus of our forthcoming research to further validate NAPS-A as a high-value functional ingredient.

## 5. Conclusions

In conclusion, NAPS-A, a food-derived bioactive polysaccharide sustainably produced via the liquid fermentation of *Naematelia aurantialba* basidiospores, exerted potent restorative effects against UVB-induced photoaging in L929 fibroblasts. These protective benefits are mediated by a synergistic mechanism that enhances antioxidant defense, suppresses inflammatory responses, and maintains ECM homeostasis. Our findings underscore the substantial translational potential of NAPS-A as a high-value functional ingredient for the development of innovative nutraceuticals and foods targeting skin health.

## Figures and Tables

**Table 1:** Primer sequences for real-time PCR.

Gene	Forward Primer (5′ to 3′)	Reverse Primer (5′ to 3′)
*GAPDH*	CATCCACTGGTGCTGCCAAGGCTGT	ACAACCTGGTCCTCAGTGTAGCCCA
*Col1a1*	ACGTCCTGGTGAAGTTGGTC	CAGGGAAGCCTCTTTCTCCT
*Col3a1*	TGGTCCTCAGGGTGTAAAGG	GTCCAGCATCACCTTTTGGT
*Tnf-α*	ATCGGTCCCAACAAGGAGGA	CTCCGCTTGGTGGTTTGCTAC

## Data Availability

The original contributions presented in this study are included in the article. Further inquiries can be directed to the corresponding author.

## References

[1] Laure Rittié G.J.F. (2002). UV-light-induced signal cascades and skin aging. Ageing Res. Rev..

[2] Gu Y., Han J., Jiang C., Zhang Y. (2020). Biomarkers, oxidative stress and autophagy in skin aging. Ageing Res. Rev..

[3] Hoel D.G., Berwick M., de Gruijl F.R., Holick M.F. (2016). The risks and benefits of sun exposure 2016. Derm.-Endocrinol..

[4] De Gruijl F.R., van Kranen H.J., Mullenders L.H.F. (2001). UV-induced DNA damage, repair, mutations and oncogenic pathways in skin cancer. J. Photochem. Photobiol. B Biol..

[5] Melo C.P.B., Saito P., Martinez R.M., Staurengo-Ferrari L., Pinto I.C., Rodrigues C.C.A., Badaro-Garcia S., Vignoli J.A., Baracat M.M., Bussmann A.J.C. (2023). Aspirin-Triggered Resolvin D1 (AT-RvD1) Protects Mouse Skin against UVB-Induced Inflammation and Oxidative Stress. Molecules.

[6] Wang J., Yuan M., Li Q., Shen C., Zhang X., Zhu C., Cen Q. (2025). Combined protection against UVB-induced photoaging by oleuropein, hydroxytyrosol, and verbascoside through modulation of inflammation, oxidative stress, and collagen homeostasis. Sci. Rep..

[7] Zúñiga-López M.C., Maturana G., Campmajó G., Saurina J., Núñez O. (2021). Determination of Bioactive Compounds in Sequential Extracts of Chia Leaf (*Salvia hispanica* L.) Using UHPLC-HRMS (Q-Orbitrap) and a Global Evaluation of Antioxidant In Vitro Capacity. Antioxidants.

[8] Qiu W.-L., Chao C.-H., Lu M.-K. (2024). Anti-inflammatory and anti–lung cancer activities of low-molecular-weight and high-sulfate-content sulfated polysaccharides extracted from the edible fungus *Poria cocos*. Int. J. Biol. Macromol..

[9] Singh A., Saini R.K., Kumar A., Chawla P., Kaushik R. (2025). Mushrooms as Nutritional Powerhouses: A Review of Their Bioactive Compounds, Health Benefits, and Value-Added Products. Foods.

[10] Lin M., Bao C., Chen L., Geng S., Wang H., Xiao Z., Gong T., Ji C., Cheng B. (2023). *Tremella fuciformis* polysaccharides alleviates UV-provoked skin cell damage via regulation of thioredoxin interacting protein and thioredoxin reductase 2. Photochem. Photobiol. Sci..

[11] Zeng Q., Zhou F., Lei L., Chen J., Lu J., Zhou J., Cao K., Gao L., Xia F., Ding S. (2017). *Ganoderma lucidum* polysaccharides protect fibroblasts against UVB-induced photoaging. Mol. Med. Rep..

[12] Lin H., Cheng K.-C., Lin J.-A., Hsieh L.-P., Chou C.-H., Wang Y.-Y., Lai P.-S., Chu P.-C., Hsieh C.-W. (2022). *Pholiota nameko* Polysaccharides Protect against Ultraviolet A-Induced Photoaging by Regulating Matrix Metalloproteinases in Human Dermal Fibroblasts. Antioxidants.

[13] Du X., Zhang Y., Mu H., Lv Z., Yang Y., Zhang J. (2015). Structural elucidation and antioxidant activity of a novel polysaccharide (TAPB1) from *Tremella aurantialba*. Food Hydrocoll..

[14] Huang G., Guo Z., Tan J.n., Xu Q., Wei C. (2025). Optimization of Ultrasonic Extraction, Functional Properties, and Antioxidant Activity of *Naematelia aurantialba* Polysaccharides. Starch-Stärke.

[15] Sun T., Xu X., Ma Y., Jiang H., Yang K., Wang R., Gu Y., Li S., Qiu Y., Sun D. (2023). Structure, rheology, and antifreeze property of the exopolysaccharide from *Naematelia aurantialba* through basidiospore fermentation. Food Hydrocoll..

[16] Zhong Y., Tan P., Lin H., Zhang D., Chen X., Pang J., Mu R. (2024). A Review of *Ganoderma lucidum* Polysaccharide: Preparations, Structures, Physicochemical Properties and Application. Foods.

[17] Sun T., Liang X., Xu X., Wang L., Xiao W., Ma Y., Wang R., Gu Y., Li S., Qiu Y. (2024). In vitro digestion and fecal fermentation of basidiospore-derived exopolysaccharides from *Naematelia aurantialba*. Int. J. Biol. Macromol..

[18] Zhou H., Li W., Pan L., Zhu T., Zhou T., Xiao E., Wei Q. (2024). Human extracellular matrix (ECM)-like collagen and its bioactivity. Regen. Biomater..

[19] Madan K., Nanda S. (2018). In-vitro evaluation of antioxidant, anti-elastase, anti-collagenase, anti-hyaluronidase activities of safranal and determination of its sun protection factor in skin photoaging. Bioorganic Chem..

[20] Papaccio F., D′Arino A., Caputo S., Bellei B. (2022). Focus on the Contribution of Oxidative Stress in Skin Aging. Antioxidants.

[21] Guo L., Dai H., Ma J., Wang J., Hua Y., Zhou L. (2021). Isolation, structure characteristics and antioxidant activity of two water-soluble polysaccharides from *Lenzites betulina*. BMC Chem..

[22] Sa-nguanpong P., Wetprasit P., Chatturong U., Chootip K., Kantip N., Tochampa W., Ruttarattanamongkol K., Bualeong T. (2025). Protective effects of Sacha inchi meal protein hydrolysate against oxidative stress and endothelial dysfunction via MDA suppression and SOD activation in L-NAME-induced hypertensive rats. Eur. J. Med. Chem. Rep..

[23] Manosalva C., Alarcón P., Grassau L., Cortés C., Hancke J.L., Burgos R.A. (2025). Andrographolide Mitigates Inflammation and Reverses UVB-Induced Metabolic Reprogramming in HaCaT Cells. Int. J. Mol. Sci..

[24] Franco A.C., Aveleira C., Cavadas C. (2022). Skin senescence: Mechanisms and impact on whole-body aging. Trends Mol. Med..

[25] Iovine B., Nino M., Irace C., Bevilacqua M.A., Monfrecola G. (2009). Ultraviolet B and A irradiation induces fibromodulin expres-sion in human fibroblasts in vitro. Biochimie.

[26] Mapoung S., Umsumarng S., Semmarath W., Arjsri P., Thippraphan P., Yodkeeree S., Limtrakul Dejkriengkraikul P. (2021). Skin Wound-Healing Potential of Polysaccharides from Medicinal Mushroom *Auricularia auricula-judae* (Bull.). J. Fungi.

[27] Zhang H., Li G., Li W., Li Y., Zhang S., Nie Y. (2024). Biochemical properties of sludge derived hydrothermal liquid products and microbial response of wastewater treatment. Process Biochem..

[28] Kiyama M., Someya Y., Sakai H., Kitamura H., Ueda M., Chigusa Y., Yonamine S., Soga S., Nanri H., Kon R. (2025). Downregulation of collagen and elastin genes in murine skin following cisplatin and vincristine treatment. Toxicol. Appl. Pharmacol..

[29] Hsiao Y., Shao Y., Wu Y., Hsu W., Cheng K., Yu C., Chou C., Hsieh C. (2023). Physicochemical properties and protective effects on UVA-induced photoaging in Hs68 cells of *Pleurotus ostreatus* polysaccharides by fractional precipitation. Int. J. Biol. Macromol..

[30] Zhang J., Yu H., Man M.Q., Hu L. (2023). Aging in the dermis: Fibroblast senescence and its significance. Aging Cell.

[31] Venkatachalam G., Surana U., Clément M.-V. (2017). Replication stress-induced endogenous DNA damage drives cellular senescence induced by a sub-lethal oxidative stress. Nucleic Acids Res..

[32] Zhang H., Davies K.J.A., Forman H.J. (2015). Oxidative stress response and Nrf2 signaling in aging. Free Radic. Biol. Med..

[33] Li S., Song Z., Liu T., Liang J., Yuan J., Xu Z., Sun Z., Lai X., Xiong Q., Zhang D. (2018). Polysaccharide from *Ostrea rivularis* attenuates reproductive oxidative stress damage via activating Keap1-Nrf2/ARE pathway. Carbohydr. Polym..

[34] Wang J., Hu S., Nie S., Yu Q., Xie M. (2016). Reviews on Mechanisms of In Vitro Antioxidant Activity of Polysaccharides. Oxid. Med. Cell. Longev..

[35] Wu J., Li P., Tao D., Zhao H., Sun R., Ma F., Zhang B. (2018). Effect of solution plasma process with hydrogen peroxide on the degradation and antioxidant activity of polysaccharide from *Auricularia auricula*. Int. J. Biol. Macromol..

[36] Yuan Q., Xie Y., Wang W., Yan Y., Ye H., Jabbar S., Zeng X. (2015). Extraction optimization, characterization and antioxidant activity in vitro of polysaccharides from mulberry (*Morus alba* L.) leaves. Carbohydr. Polym..

[37] Wang M., Ma J. (2019). Effect of NGR1 on the atopic dermatitis model and its mechanisms. Open Med..

[38] Liao J., Liu Z., Wu S. (2024). *Hypericum sampsonii* ameliorates radiodermatitis by inhibiting NLRP3 inflammasome activation. Ski. Res. Technol..

[39] Ruscinc N., Massarico Serafim R.A., Almeida C., Rosado C., Baby A.R. (2024). Challenging the safety and efficacy of topically applied chlorogenic acid, apigenin, kaempferol, and naringenin by HET-CAM, HPLC-TBARS-EVSC, and laser Doppler flowmetry. Front. Chem..

